# Progression patterns, resistant mechanisms and subsequent therapy for ALK-positive NSCLC in the era of second-generation ALK-TKIs

**DOI:** 10.1186/s12967-024-05388-0

**Published:** 2024-06-20

**Authors:** Lige Wu, Zihua Zou, Yan Li, Xuezhi Hao, Jianming Ying, Junling Li, Puyuan Xing

**Affiliations:** 1 Department of Medical Oncology, National Cancer Center/National Clinical Research Center for Cancer/Cancer Hospital, Chinese Academy of Medical Sciences and Peking Union Medical College, No 17 Panjiayuan Nanli, Chaoyang district, Beijing, 100021 P.R. China; 2 Department of Medical Oncology, Clinical Oncology School of Fujian Medical University, Fujian Cancer Hospital, Fuzhou, 350014 P.R. China; 3 Department of Pathology, National Cancer Center/National Clinical Research Center for Cancer/Cancer Hospital , Chinese Academy of Medical Sciences and Peking Union Medical College, No 17 Panjiayuan Nanli, Chaoyang district, Beijing, 100021 P.R. China

**Keywords:** ALK, NSCLC, Real-world study, Resistance

## Abstract

**Background:**

In the era of second-generation ALK tyrosine kinase inhibitors (ALK-TKIs), there was a paucity of data regarding the progression patterns, resistant mechanisms, and subsequent therapeutic approaches for ALK-positive (ALK^+^) non-small cell lung cancer (NSCLC).

**Methods:**

Patients with advanced ALK^+^ NSCLC were retrospectively selected from our center. Cohort 1 consisted of patients who experienced disease progression after receiving first-line alectinib treatment (*n* = 20), while Cohort 2 included patients who progressed following sequential treatment with crizotinib and second-generation ALK-TKIs (*n* = 53). Oligo-progression was defined as the occurrence of disease progression in no more than three lesions. Symptomatic progression was determined when patients developed new symptoms or experienced worsening of pre-existing symptoms during radiological progression.

**Results:**

The incidence of central nervous system (CNS) progression and symptomatic CNS progression was significantly lower in Cohort 1 compared to patients treated with crizotinib, with rates of 15.0% vs. 56.6% (*p* = 0.002) and 5.0% vs. 32.1% (*p* = 0.016), respectively. A total of 60.3% (44/73) patients underwent repeated biopsy and next-generation sequencing subsequent to the second-generation ALK-TKI resistance, with secondary mutation in ALK kinase domain emerging as the predominant mechanism of resistance (56.8%). Local therapy was applied to 50% of oligo-progression cases. Subsequent ALK-TKIs demonstrated significantly prolonged progression-free survival (PFS) (8.6 m vs. 2.7 m, *p* = 0.021, HR = 0.43, 95%CI: 0.15–0.85) and long-term overall survival (OS) (NA vs. 11.9 m, *p* = 0.132, HR = 0.50, 95%CI: 0.18–1.25) in patients harboring ALK resistance mutations, compared to those without such mutations. For patients without ALK-resistant mutations following progression on second-generation ALK-TKIs, there was no statistically significant difference in survival outcomes between subsequent chemotherapy or alternative ALK-TKI treatments.

**Conclusions:**

First-line alectinib demonstrated superior efficacy in protecting the CNS compared to crizotinib. For patients with ALK-resistant mutations following the resistance to second-generation ALK-TKIs, appropriate sensitive ALK-TKI should be administered; for those without such mutations, the selection of chemotherapy or third-generation ALK-TKI should be based on the patient’s overall physical health and personal preferences.

**Supplementary Information:**

The online version contains supplementary material available at 10.1186/s12967-024-05388-0.

## Introduction

Since the identification of anaplastic lymphoma kinase (ALK) rearrangement in non-small cell lung cancer (NSCLC) in 2007 [1], significant advancements have been made in this field. Patients with advanced ALK-positive (ALK^+^) NSCLC have experienced substantial survival benefits due to the development of multiple generations of ALK inhibitors [2]. However, resistance inevitably emerges during tumor evolution. It is important to note that while clinical efficacy remains the primary endpoint in randomized controlled trials (RCTs), progression patterns, mechanisms of resistance, and subsequent therapies are not extensively discussed within this context. Consequently, many real-world studies and translational medicine research have emerged to solve these problems.

Prior studies have demonstrated that approximately 50% of patients experience central nervous system (CNS) progression following crizotinib treatment, a first-generation ALK inhibitor [3]. Whereas alectinib demonstrates superior efficacy in the prevention of CNS metastasis, findings from the J-ALEX study indicate that treatment with alectinib resulted in an 84% reduction in the risk of CNS progression compared to crizotinib among patients with existing CNS metastases [4]. A pooled analysis revealed that, for patients with baseline CNS metastasis, the objective response rate (ORR) within the CNS was 42.6% (95% CI, 34.2–51.4%), and the median duration of response within the CNS was 11.1 months (95% CI, 10.3 months to not evaluable) [5]. However, the incidence of CNS progression upon resistance to second-generation ALK inhibitors remains unknown. Furthermore, with significant advancements in radiotherapy and interventional therapy, there is increasing emphasis on local treatment for oligo-metastases and oligo-progression in clinical practice [6]. Nevertheless, the incidence of oligo-progression subsequent to ALK inhibitor therapy remains unclear.

Furthermore, previous studies have demonstrated that resistance mechanisms to ALK inhibitors can be broadly categorized into three groups: inadequate drug exposure in CNS, ALK-dependent resistant mechanisms, and ALK-independent resistant mechanisms (including bypass activation and pathological transformation) [7, 8]. The dominant mechanism of resistance to crizotinib is inadequate drug exposure in CNS due to its low penetration rate across the blood-brain barrier (BBB) (with a cerebrospinal fluid-to-plasma drug concentration ratio of 0.26%) [9]. On the other hand, secondary mutations in ALK kinase domain are primarily responsible for resistance to second-generation ALK inhibitors [10]. While repeated biopsy undoubtedly aids in identifying resistance mechanisms, limited real-world data exists regarding optimal timing for re-biopsy.

In terms of subsequent therapy, the preferred option for resistance to crizotinib is sequential treatment with second-generation ALK inhibitors [11]. For patients who experience progression after receiving second-generation ALK inhibitors, clinicians typically select an appropriate ALK inhibitor based on the specific mutation site in ALK kinase domain (e.g., ceritinib for I1171N sensitivity, alectinib for F1174L sensitivity, brigatinib for V1180L sensitivity) [12, 13]. However, limited knowledge exists regarding the optimal subsequent therapy for patients without ALK resistance mutations.

Currently, the third-generation ALK-TKI lorlatinib has obtained approval for the treatment of ALK^+^ NSCLC in multiple countries. The latest findings from the CROWN study demonstrate a 5-year progression-free survival (PFS) rate of up to 60% for first-line lorlatinib, showcasing elevated intracranial ORR and enhanced capacity to inhibit brain metastasis [14, 15]. The unique structure of lorlatinib enhances its ability to penetrate the blood-brain barrier and delay resistance development. The design of lorlatinib incorporates a macrocyclic amide structure, demonstrating enhanced metabolic stability, reduced molecular weight (~ 400), and increased lipophilicity. These characteristics are advantageous in reducing P-glycoprotein mediated drug efflux, enhancing the ability of lorlatinib to traverse the blood-brain barrier, and delaying the onset of drug resistance [16]. A Phase II study revealed that lorlatinib exhibited systemic and intracranial activity in patients resistant to crizotinib or other ALK inhibitors, indicating its potential efficacy [17]. However, for patients resistant to second-generation ALK-TKIs, there is currently insufficient evidence to determine the optimal choice between chemotherapy, third-generation ALK-TKI, or other ALK-TKI therapies.

Based on these questions, we conducted a retrospective study to investigate the progression patterns, mechanisms of resistance, and subsequent therapeutic approaches for patients with ALK^+^ NSCLC in the era of second-generation ALK inhibitors.

## Methods

### Patient selection

Our research was a single-center retrospective study. Patients with advanced ALK^+^ NSCLC who exhibited disease progression subsequent to treatment with second-generation ALK-TKIs were enrolled and subsequently stratified into two distinct cohorts. Cohort 1 comprised patients who exhibited disease progression subsequent to initial treatment with alectinib, whereas Cohort 2 consisted of patients who encountered progression following sequential therapy involving crizotinib and second-generation ALK inhibitors. The study protocol required that all enrolled patients undergo a comprehensive radiological examination at baseline and subsequently receive regular radiological evaluations every 2–3 months during the follow-up period. Patients with a second primary tumor other than lung cancer were excluded.

### Data extraction

The demographic and clinical characteristics were meticulously documented. Repetitive biopsy results were also documented during the progression of ALK inhibitors. The next-generation sequencing (NGS) results obtained from re-biopsy specimens of ALK-TKI resistant patients were also collected and subjected to analysis. The detailed sequencing data of 15 patients in our hospital (Burning Rock, 168 panel) was available, while information regarding resistant mechanisms in other patients was obtained from Electronic Medical Records (EMR) or other biogenetics firms. The authors conducted a comprehensive review of imaging data to assess the treatment response, while survival information was obtained through meticulous examination of clinical records or diligent follow-up via telephone by investigators. The data cut-off date for this study was February 28, 2024. In the event of a patient being lost to follow-up on February 28, 2024, the most recent follow-up data available was considered as the designated cut-off date.

### Evaluation criteria and study endpoints

The radiological assessment of intracranial and extracranial lesions was conducted in accordance with the Response Evaluation Criteria in Solid Tumors version 1.1 (RECIST 1.1). The study endpoints encompassed the assessment of disease progression patterns, timing of repeated biopsy, identification of potential resistant mechanisms for second-generation ALK-TKIs, and evaluation of the efficacy of subsequent treatments. Oligo-progression was defined as disease progression in no more than three lesions, while progression in pulmonary lymphangitis, pleural/serous effusion or leptomeningeal metastases was considered extensive-progression. Patients who exhibited new symptoms or experienced exacerbation of pre-existing symptoms during radiological progression were classified as symptomatic progression. The analysis of resistance mechanisms included patients who underwent repeated biopsy upon resistance to second-generation ALK-TKIs. The PFS was defined as the duration from the initiation of anti-cancer treatment to the initial radiological evidence of disease progression. Overall survival (OS) was defined as the duration from the initiation of anti-cancer treatment until death, irrespective of the cause.

### Statistical analysis

Statistical analysis was performed using SPSS 26.0 statistical software (Inc., Chicago, IL, USA). Descriptive statistics were used to present the distribution of patients and baseline demographic/clinical characteristics. Categorical data were compared using Pearson’s χ2 test, while continuous data were compared using Student’s t tests. Survival curves were estimated using the Kaplan-Meier method, and differences in variables were assessed with the log-rank test. Cox’s proportional hazard model was employed to estimate the hazard ratio (HR) and its corresponding 95% confidence interval (CI) for the covariate of interest. The statistical significance was determined by a two-sided p-value < 0.05.

## Results

### Baseline characteristics

A total of 20 patients were enroll in cohort 1 and 53 patients in cohort 2. All 53 patients in cohort 2 were sequentially treated with second-generation ALK-TKIs after crizotinib resistance. In the post-crizotinib stage of cohort 2, aletinib was administered to 33 patients, brigatinib to 10 patients, ceritinib to 7 patients, WX-0593 to 2 patients, and ensartinib to 1 patient.The baseline characteristics prior to the initiation of ALK-TKI treatment were presented in Table 1. It is noteworthy that one patient exhibited squamous-cell carcinoma (SCC) transformation subsequent to crizotinib progression. Furthermore, a considerable proportion of patients developed central nervous system (CNS) metastases upon resistance to crizotinib treatment (20.7%→60.4%).

### Progression pattern

Table 2 illustrates the progression patterns observed in different cohorts. Our findings demonstrated a higher susceptibility to central nervous system (CNS) progression in patients receiving first-line crizotinib compared to those treated with first-line alectinib, both in the overall population (56.6% vs. 15.0%, *p* = 0.002) and among patients with baseline CNS metastases (72.7% vs. 16.7%, *p* = 0.050), as well as those without baseline CNS metastases (52.4% vs. 14.3%, *p* = 0.015). In the post-crizotinib phase, all patients were subsequently treated with second-generation ALK-TKIs. Ceritinib was administered to seven patients, while other different second-generation ALK-TKIs were chosen by 46 patients. During the second-generation ALK-TKI treatment phase, there was a significant increase in the incidence of CNS progression following administration of ceritinib therapy, observed across both the entire study population (85.7% vs. 39.1%, *p* = 0.038), as well as among patients with or without pre-existing CNS metastases (100% vs. 51.7%, *p* = 0.238; 75% vs. 17.6%, *p* = 0.053 respectively). Additionally, oligo-progression occurred in approximately 30% of patients following ALK-TKI treatment (Cohort 1: 30%, Cohort 2 crizotinib stage: 34.6%, Cohort 2 post-crizotinib stage: 30.2%).

**Table 1:** Baseline characteristics of patients involved in the study

	Cohort 1	Cohort 2 Crizotinib stage	Cohort 2 Post-crizotinib stage
Number of cases	20	53	53
Gender			
Male Female	10 (50%) 10 (50%)	30 (56.6%) 23 (43.4%)	30 (56.6%) 23 (43.4%)
Median age (range) < 65 ≥ 65	52 (33–76) 18 (90%) 2 (10%)	49 (20–68) 50 (94.3%) 3 (5.7%)	50 (21–69) 49 (92.4%) 4 (7.6%)
Pathology			
Adenocarcinoma Other type	20 (100%) 0	52 (98.1%) 1 (1.9%)	51 (96.2%) 2 (3.8%)
Smoking history			
Smoker Never smoker	6 (30%) 14 (70%)	15 (28.3%) 38 (71.7%)	15 (28.3%) 38 (71.7%)
ECOG			
0–1 ≥ 2	9 (45%) 11 (55%)	41 (77.4%) 12 (22.6%)	31 (58.5%) 22 (41.5%)
Distant metastasis			
Yes No	20 (100%) 0	53 (100%) 0	53 (100%) 0
CNS metastases			
Yes No	6 (30%) 14 (70%)	11 (20.7%) 42 (79.3%)	32 (60.4%) 21 (39.6%)

**Table 2:** Patterns of ALK-TKI resistance in the real world

	Alectinib *n* = 20	Crizotinib *n* = 53	p1	Ceritinib *n* = 7	Other ALK-TKIs *n* = 46	p2
CNS progression rate in all populations	15.0%	56.6%	0.002	85.7%	39.1%	0.038
CNS progression rate in patients with baseline CNS metastases	16.7%	72.7%	0.05	100.0%	51.7%	0.238
CNS progression rate in patients without baseline CNS metastases	14.3%	52.4%	0.015	75.0%	17.6%	0.053
Symptomatic progression in all populations	40.0%	50.9%	0.442	28.6%	36.9%	1
Symptomatic CNS progression in all populations	5.0%	32.1%	0.016	28.6%	8.7%	0.174

*p*1: Alectinib versus Crizotinib; *p*2: Ceritinib versus Other ALK-TKIs. The statistical significance between the two groups was determined at a level of *p* < 0.05

Symptomatic progression was observed in nearly half of the patients upon resistance to first-line alectinib or crizotinib treatment (40.0% vs. 50.9%, *p* = 0.442); however, patients receiving crizotinib exhibited a higher susceptibility to symptomatic CNS progression compared to those treated with alectinib (32.1% vs. 5.0%, *p* = 0.016). In Cohort 2, approximately 30% of patients experienced symptomatic progression after treatment with ceritinib or other second-generation ALK inhibitors during the post-crizotinib stage (28.6% vs. 36.9%, *p* = 1), while those who received ceritinib appeared to have a higher likelihood of developing symptomatic CNS progression (28.6% vs. 8.7%, *p* = 0.174).

### Re-biopsy and resistance mechanism

**Fig. 1:**
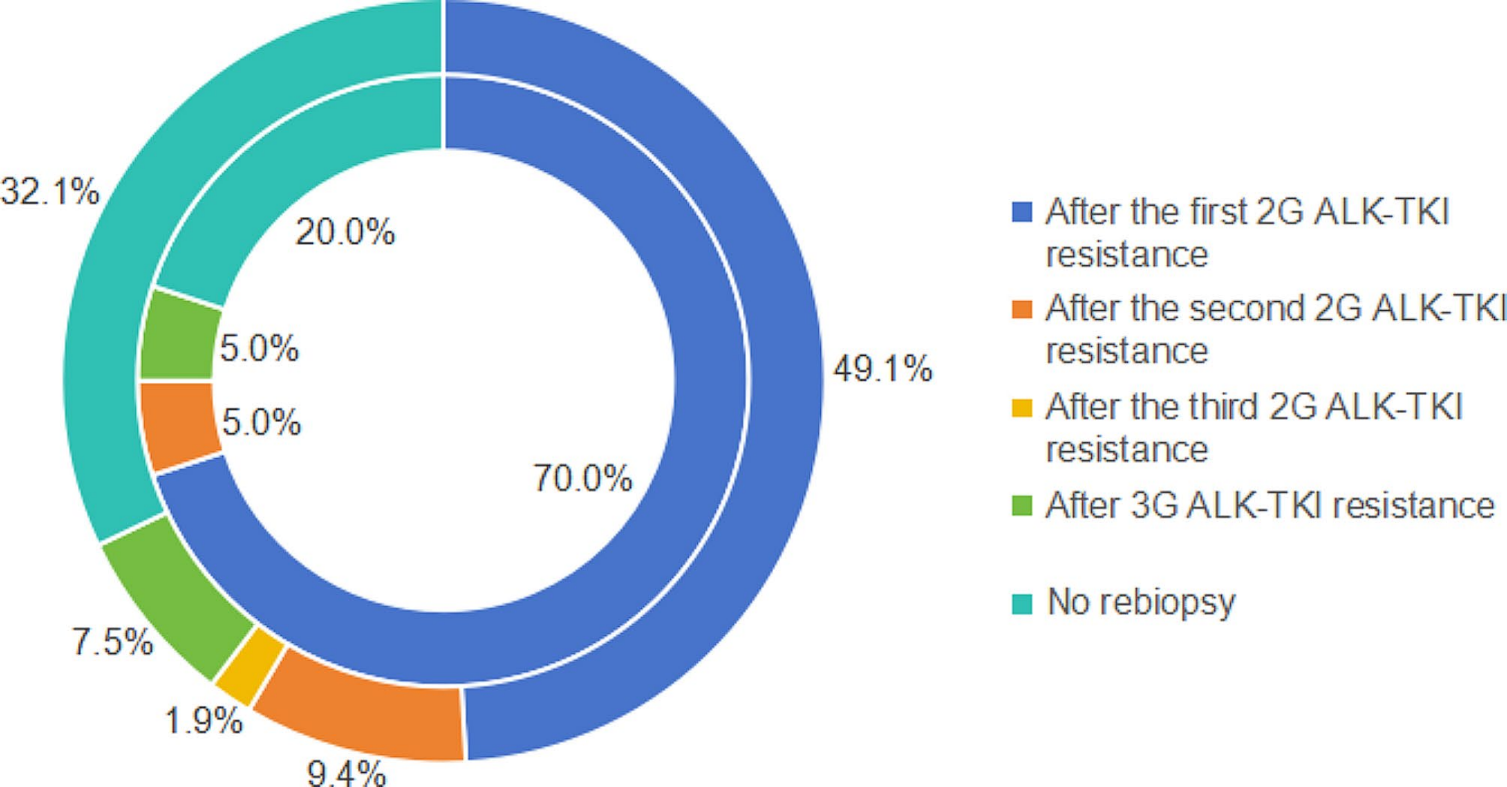
Re-biopsy timing in patients with ALK mutations receiving different treatment modalities. The inner layer is the proportion of different re-biopsy timing for cohort 1 patients (*n* = 20). The outer layer is the proportion of different re-biopsy timing for cohort 2 patients (*n* = 53) in the post-crizotinib stage. During the crizotinib phase in cohort 2, only 1 patient (1.9%) underwent re-biopsy after developing resistance. 2G, second generation; 3G, third generation

Only one patient underwent re-biopsy upon resistance to crizotinib, whereas 54.8% (40/73) of patients underwent repeated biopsy following resistance to second-generation ALK-TKI treatment. The timing of re-biopsy is illustrated in Fig. 1. A total of 47 individuals underwent re-biopsies following the resistance to second-generation ALK-TKI treatment. Among them, two cases exhibited inadequate tissue samples obtained through biopsy puncture, while one case utilized a liquid biopsy approach. Consequently, a total of 44 patients underwent subsequent tissue next-generation sequencing (NGS) and were enrolled in the investigation on resistance mechanisms to second-generation ALK-TKI therapy (Fig S1).

**Fig. 2:**
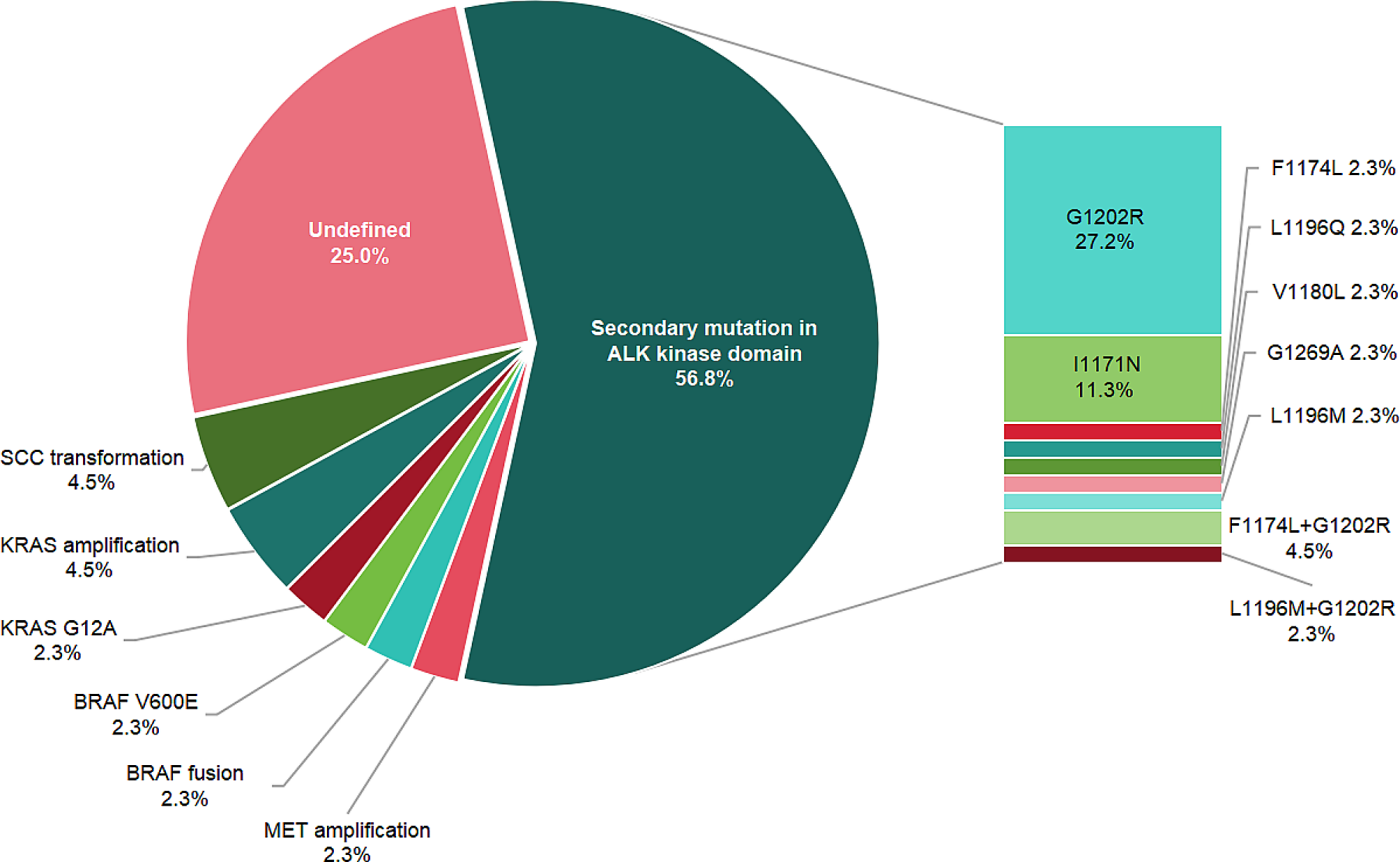
Potential mechanism of second-generation ALK-TKI resistance. The NGS results following second-generation ALK-TKI resistance in 44 patients were subjected to statistical analysis, revealing that secondary mutations in ALK kinase domains (25/44, 56.8%) represent the primary mechanism of resistance. And G1202R mutation was the most common mutation site (12/25, 48.0%) in ALK kinase domains

Our findings demonstrate that the dominant mechanism of second-generation ALK-TKI resistance is secondary mutation in the ALK kinase domain (56.8%, 25/44), with the G1202R mutation being the most prevalent site (27.2%, 12/44) (Fig. 2). Other potential mechanisms of resistance include MET amplification (*n* = 1, copy number = 4.5), AKAP9-BRAF fusion (*n* = 1), BRAF V600E mutation (*n* = 1), KRAS G12A mutation (*n* = 1), KRAS amplification (*n* = 2, copy numbers 2.1 and 3.4 respectively), and SCC transformation (*n* = 2).

### Subsequent treatment after second-generation ALK-TKI resistance

In a real-world setting, local treatment was administered in approximately 50% (20/40) of cases with oligo-progression, resulting in an extension of the duration of previous targeted therapy by around 6.4 months (95%CI: 5.4–7.1 months). The treatment status of patients in cohort 1 and 2 following the development of resistance to second-generation ALK-TKI is summarized in Table 3.

**Table 3:** Treatment options after second-generation ALK-TKI resistance in the real world

	Cohort 1 *n* = 20 13 dead	Cohort 2 *n* = 53 28 dead	All populations *n* = 73 41 dead
≥ 1 line of subsequent treatment			
Yes No	18 (90%) 2 (10%)	48 (90.6%) 5 (9.4%)	66 (90.4%) 7 (9.6%)
≥ 1 line of subsequent ALK-TKI			
Yes No	17 (85%) 3 (15%)	44 (83.0%) 9 (17.0%)	61 (83.6%) 12 (16.4%)
Subsequent third-generation ALK-TKI			
Yes No	11 (55%) 9 (45%)	29 (54.7%) 24 (45.3%)	40 (54.8%) 33 (45.2%)
≥ 1 line of chemotherapy			
Yes No	12 (60%) 8 (40%)	23 (43.4%) 30 (56.6%)	35 (47.9%) 38 (52.1%)
≥ 1 line of chemotherapy in dead group			
Yes No	8 (61.5%) 5 (38.5%)	15 (53.6%) 13 (46.4%)	23 (56.1%) 18 (43.9%)

In general, more than 80% of the overall population received at least one line of alternative ALK-TKI subsequent to the progression of their initial second-generation ALK-TKI. Over half of the patients had received treatment with third-generation ALK-TKI agents. Furthermore, a total of 35 patients (47.9%) received first-line or posterior platinum-doublet chemotherapy following the initial development of second-generation ALK-TKI resistance.

### Efficacy of sequential second-generation ALK-TKIs following crizotinib resistance

**Fig. 3:**
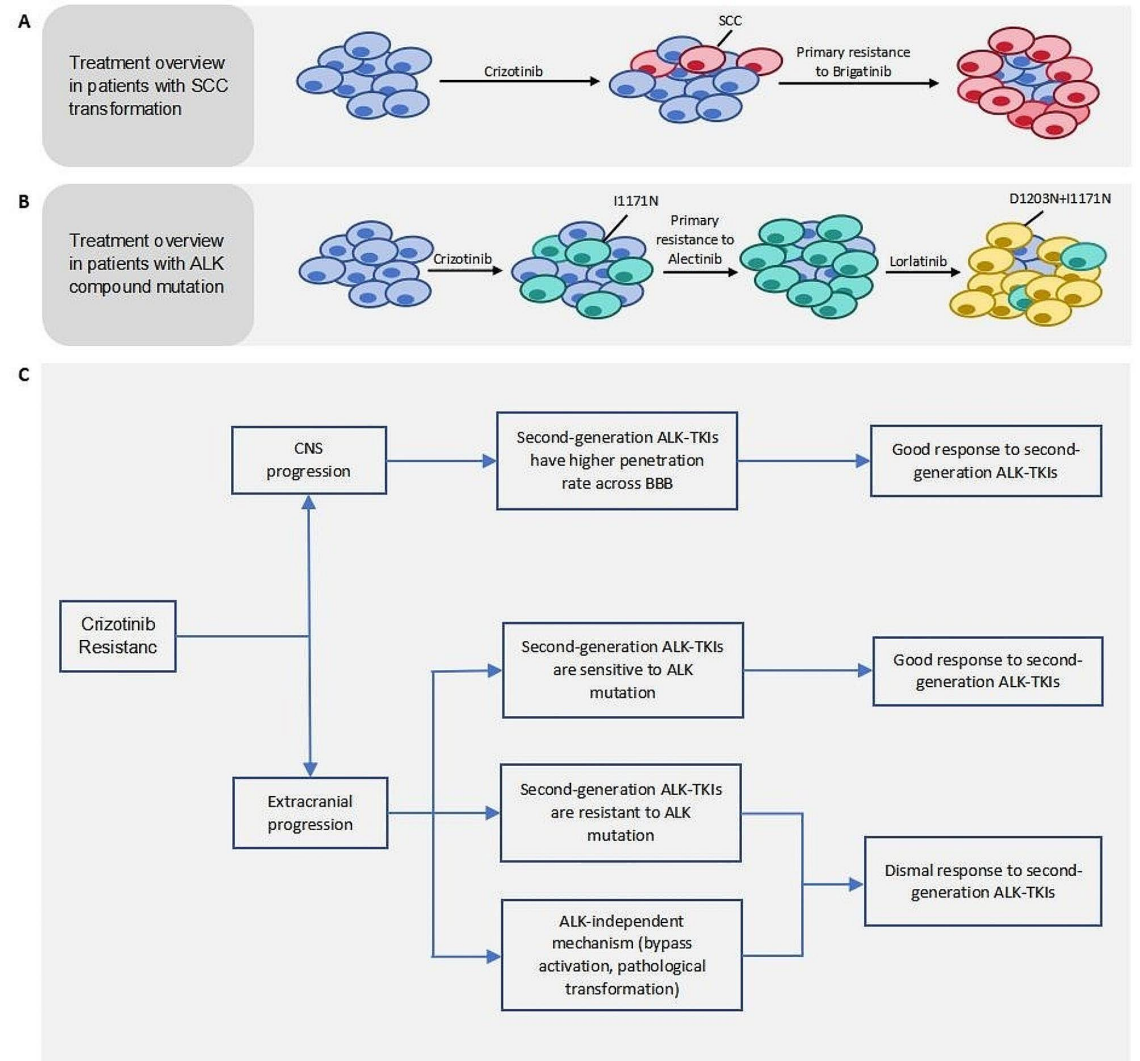
Second-generation ALK-TKI primary resistance models and therapeutic strategies after crizotinib resistance. (**A**) Second-generation ALK-TKI resistance model induced by SCC transformation. (**B**) Second-generation ALK-TKI resistance model induced by ALK compound mutation. (**C**) Prediction to the efficacy of sequential treatment of second-generation ALK-TKI. BBB, blood-brain barrier

In our study, we observed an intriguing phenomenon: primary resistance to sequential second-generation ALK-TKI was exclusively observed in patients with extracranial progression subsequent to crizotinib treatment. The cohort 1 exhibited two prototypical instances. One patient was administered brigatinib treatment subsequent to developing resistance to crizotinib; however, the PFS duration was less than 2 months. Subsequent re-biopsy revealed the presence of new squamous cell carcinoma components within the tumor, while ALK resistance mutations were not detected through NGS. These findings suggest that primary resistance to second-generation ALK-TKI in this patient may be attributed to pathological type transformation (Fig. 3A). The other patient exhibited primary resistance to alectinib subsequent to crizotinib treatment (no repeated biopsy was performed upon progression of alectinib and crizotinib). However, subsequent administration of lorlatinib resulted in a favorable response. Ultimately, the presence of compound ALK mutations (I1171N + D1203N) was confirmed at the point of lorlatinib resistance. Therefore, we hypothesized that the emergence of the I1171N mutation in ALK kinase domain occurred subsequent to crizotinib resistance and resulted in primary resistance to alectinib. And the occurrence of D1203N co-mutation contributed to the resistance of third generation ALK-TKI (Fig. 3B).

In general, second-generation ALK-TKI with enhanced CNS penetration rates are expected to exhibit significant efficacy in patients who only experience CNS progression upon resistance to crizotinib. However, for patients with extracranial progression, re-biopsy and NGS may still be necessary as second-generation ALK-TKI is not a panacea under such circumstances. In this study, we present a comprehensive analysis of various mechanisms underlying crizotinib resistance and propose effective strategies to overcome it (Fig. 3C).

### Efficacy of subsequent therapy following resistance to second-generation ALK-TKIs

**Fig. 4:**
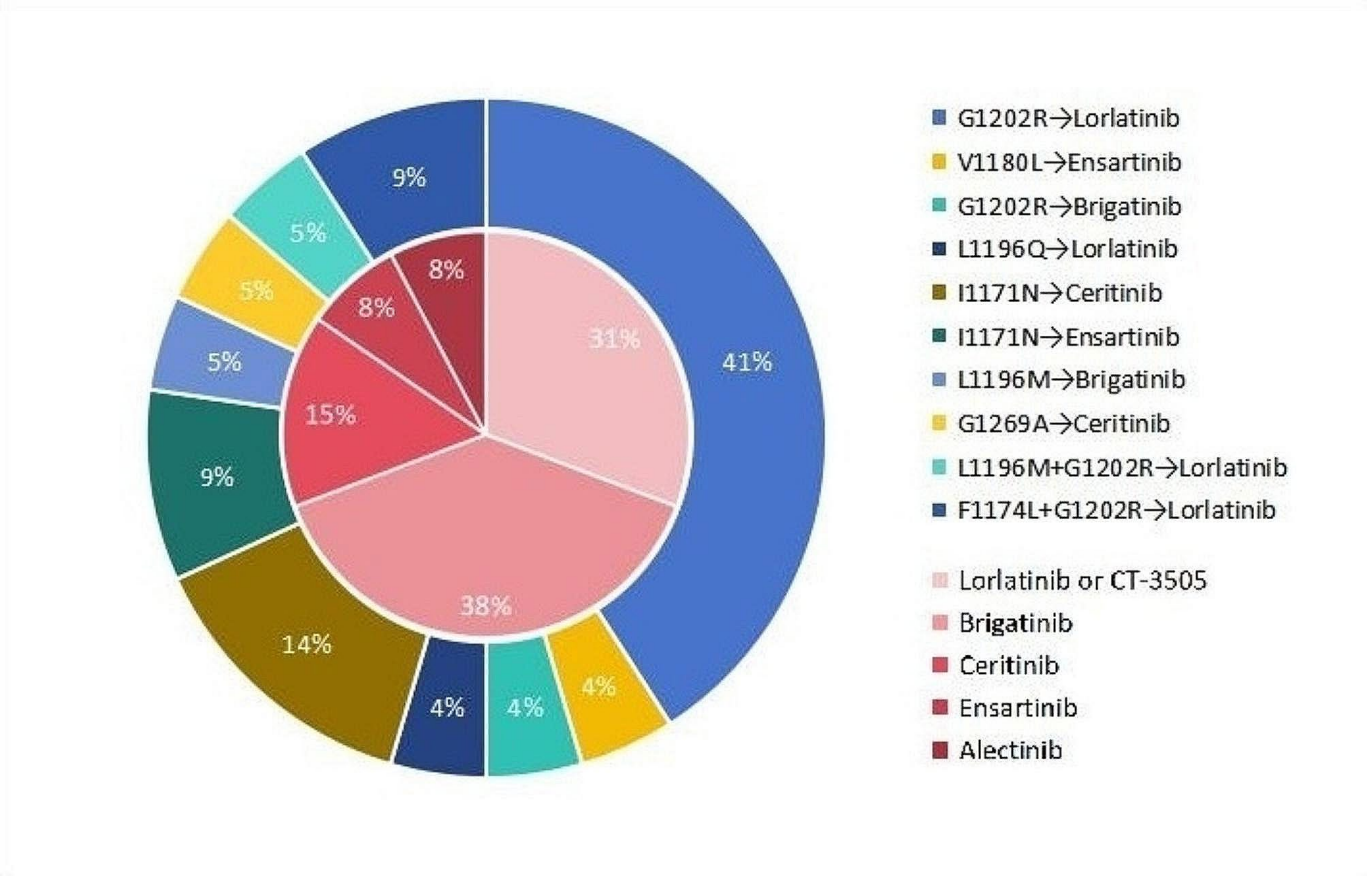
Readministration of ALK-TKIs in patients following re-biopsy. The outer layer is subsequent TKI drugs for patients with ALK-resistant mutations (*n* = 22). And the inner layer is subsequent TKI drugs for patients without ALK-resistant mutations (*n* = 13)

**Fig. 5:**
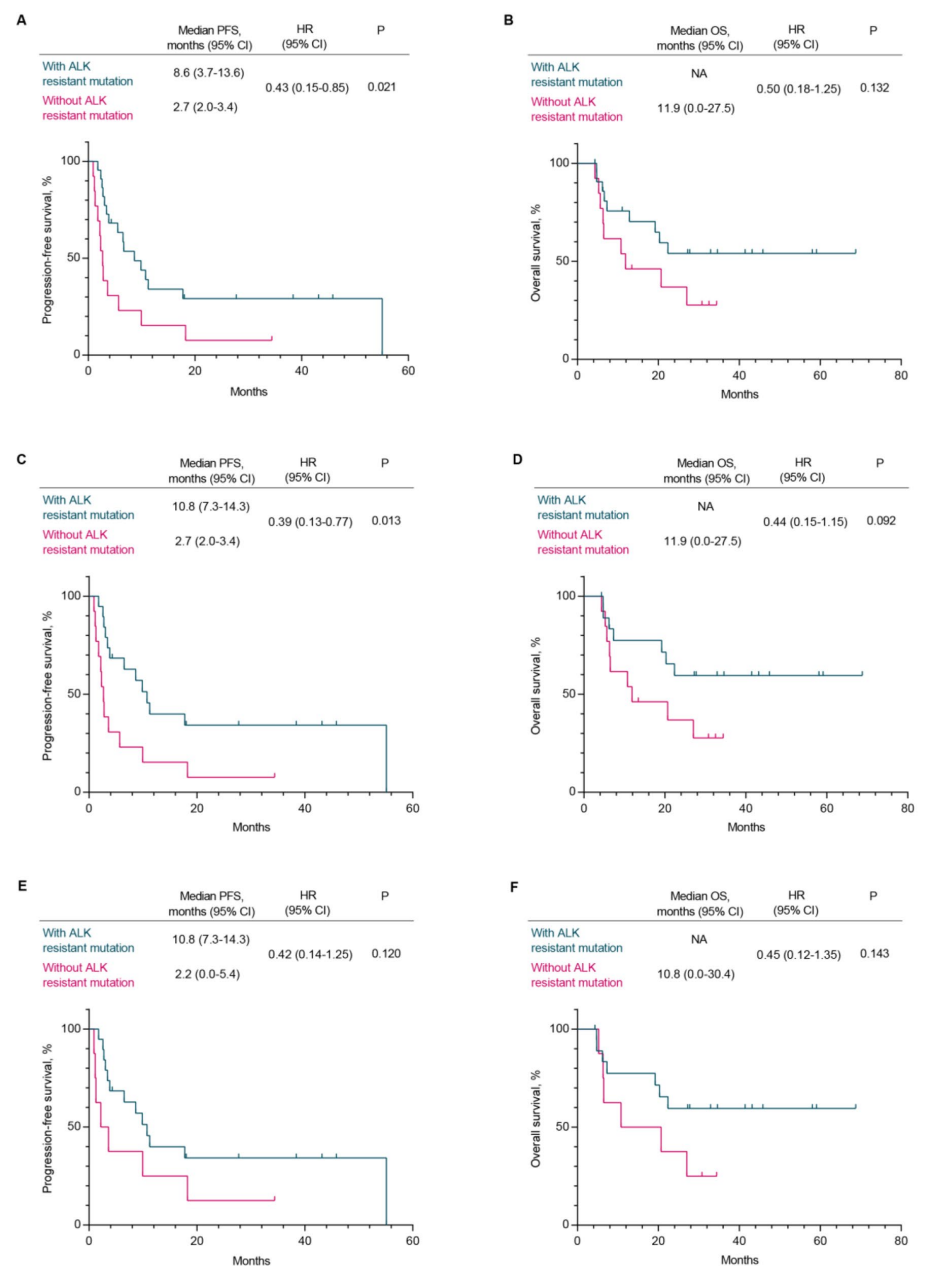
Survival outcomes in patients treated with other ALK-TKIs after second-generation ALK-TKI resistance. (**A, B**) PFS and OS in patients with (*n* = 22) and without ALK-resistant mutations (*n* = 13). (**C, D**) After excluding patients with ALK compound mutations (*n* = 3), the PFS and OS were analyzed again in the two groups. (**E, F**) In the group without ALK-resistant mutations, only include patients who have received sequential third-generation ALK-TKI treatment. PFS and OS in patients with (*n* = 19) and without ALK-resistant mutations (*n* = 8)

In this study, the majority of second-generation ALK-TKI resistant patients were subsequently administered alternative ALK-TKI treatments, as depicted in Fig. 4. Among patients with second-generation ALK-TKI resistance, a total of 35 patients were subsequently administered alternative ALK-TKI therapies, comprising 22 individuals harboring secondary mutation in ALK kinase domain and 13 individuals lacking ALK resistance mutations. The researchers conducted a long-term follow-up on these patients and provided updated survival data as of February 28, 2024. In our research, patients who developed secondary mutation in ALK kinase domain exhibited a significantly prolonged PFS when treated with alternative ALK-TKI compared to those without such mutations (8.6 m vs. 2.7 m, *p* = 0.021, HR = 0.43, 95%CI: 0.15–0.85) (Fig. 5A). For OS, although there was initially minimal disparity between the two groups, patients harboring secondary mutation in ALK kinase domain exhibited prolonged median OS in the long run (NA vs. 11.9 m, *p* = 0.132, HR = 0.50, 95%CI: 0.18–1.25) (Fig. 5B). Given the frequent resistance of lorlatinib in patients with ALK compound mutation observed in clinical practice, we excluded individuals with ALK compound mutation (*n* = 3) from our study and subsequently reanalyzed the data. In the group of patients with secondary mutation in ALK kinase domain, the PFS was 10.8 months, while the OS remained not achieved. The median PFS and OS of patients without ALK resistant mutations were 2.7 months and 11.9 months, respectively (Fig. 5C, D). For patients lacking ALK resistant mutations, third-generation ALK-TKI may be the most preferable targeted therapy; however, even with this treatment approach, both PFS and OS remain inferior to those observed in individuals harboring ALK kinase domain mutations (Fig. 5E, F). These findings suggest that patients lacking secondary mutation in ALK kinase domain encounter a bottleneck following the resistance to second-generation ALK-TKIs, and the current third-generation ALK-TKI fails to address all instances of second-generation ALK-TKI resistance.

**Fig. 6:**
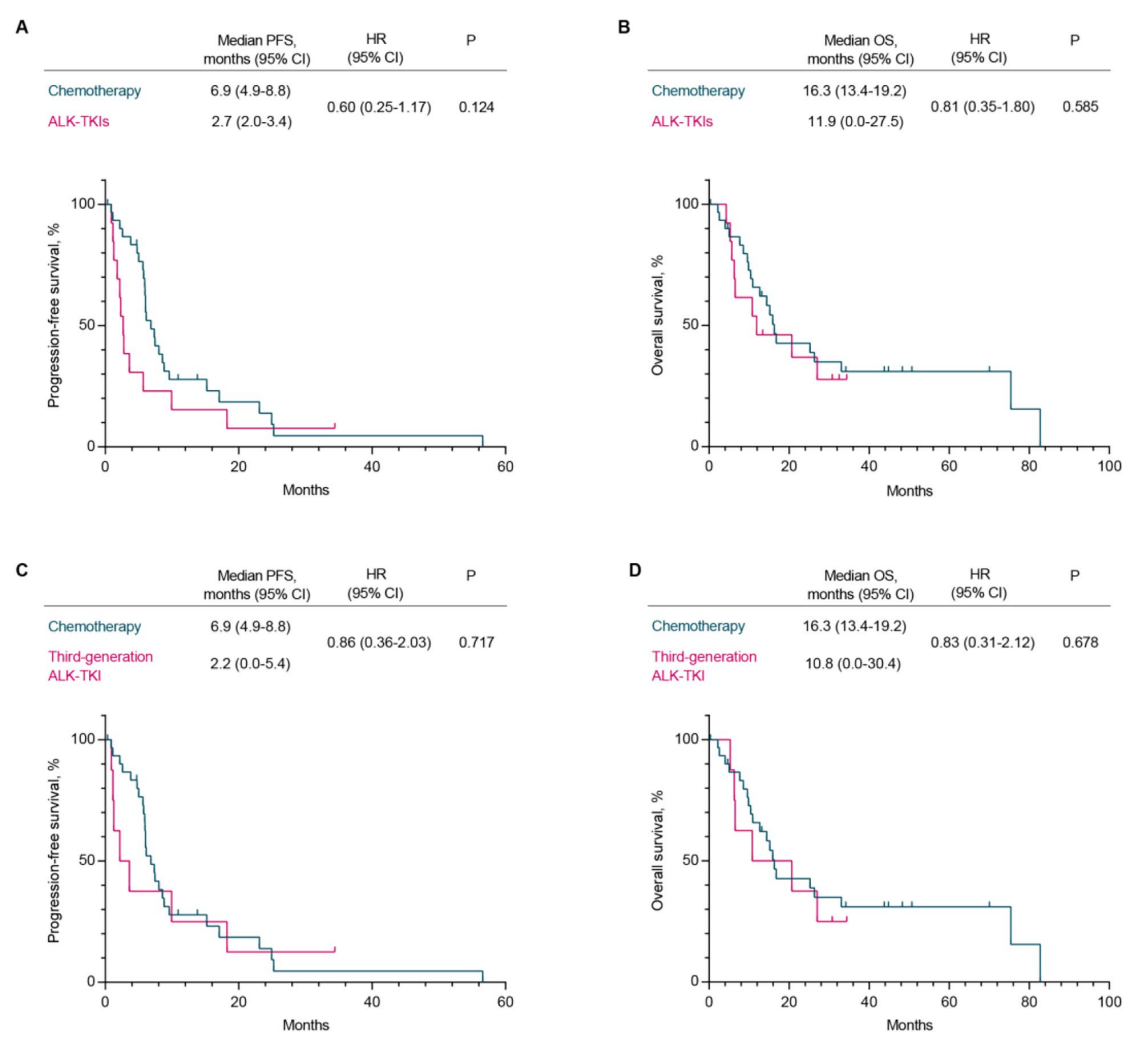
Efficacy of follow-up chemotherapy or ALK-TKIs in patients without ALK-resistant mutations. (**A.B**) PFS and OS comparison between platinum-based chemotherapy (*n* = 31) and subsequent ALK-TKIs (*n* = 13) for patients without ALK resistance mutation. (**C.D**) PFS and OS comparison between platinum-based chemotherapy (*n* = 31) and subsequent third-generation ALK-TKIs (*n* = 8) for patients without ALK resistance mutation

To identify the optimal therapeutic approach for patients lacking ALK resistant mutations following resistance to second-generation ALK-TKIs, we conducted the subsequent investigation. In clinical settings, patients who fail targeted therapies often choose chemotherapy as an alternative treatment option. In this study, a total of 31 patients opted for chemotherapy following the failure of second-generation ALK-TKIs, while 13 patients without ALK resistant mutations were subsequently treated with alternative ALK-TKIs. The long-term follow-up revealed no statistically significant difference in survival outcomes between the two cohorts. The PFS was 6.9 months in chemotherapy group and 2.7 months in ALK-TKI group, with a hazard ratio (HR) of 0.6 (95%CI: 0.25–1.17) and no statistical significance (*p* = 0.124) (Fig. 6A). The OS was 16.3 months in chemotherapy group and 11.9 months in ALK-TKI group, with a hazard ratio (HR) of 0.81 (95%CI: 0.35–1.80) and no statistical significance (*p* = 0.585) (Fig. 6B). Even when third-generation ALK-TKI was administered to patients without ALK resistant mutations, there were still no significant differences observed in PFS and OS compared to the chemotherapy group (Fig. 6C, D). Totally, the median PFS of chemotherapy was higher than that of other ALK-TKIs following resistance to second-generation ALK-TKIs, albeit lacking statistical significance. This implies that chemotherapy might be a more favorable treatment option for patients in optimal physical condition.

These findings suggest that in patients with ALK resistance mutations, alternative ALK-TKI agents should be prioritized; while in patients without ALK resistance mutations, the choice between chemotherapy or ALK-TKI treatment should be based on the patient’s ECOG PS score and adverse events. However, it must be noted that the majority of patients who received chemotherapy after second-generation ALK-TKI resistance did not undergo secondary biopsies and NGS, and whether they have secondary mutation in the ALK kinase domain is unknown. Therefore, for patients with second-generation ALK-TKI resistance and without ALK resistant mutations, the choice of third-generation ALK-TKI or chemotherapy needs to be confirmed by standardized head-to-head studies.

## Discussion

Patients with advanced ALK^+^ NSCLC have derived significant survival benefits from multiple generations of ALK inhibitors. Despite substantial progress in this field, the challenge of resistance to anticancer treatment remains formidable. While clinical efficacy of anticancer treatment has traditionally been the primary focus in randomized controlled trials (RCTs), there is often limited discussion on cancer progression patterns, resistant mechanisms, and subsequent treatments within this context. Therefore, real-world studies and translational medical research have been designed and implemented to address these key issues.

In our research, we made several noteworthy findings that could serve as a valuable reference for clinical practice. Firstly, in terms of the progression pattern, our study revealed a significantly lower incidence of CNS progression in patients receiving first-line alectinib compared to those treated with crizotinib. Additionally, this study investigated the occurrence of symptomatic progression which had not been reported previously. Although no significant difference was observed in the incidence of symptomatic progression between these two cohorts, patients treated with first-line alectinib exhibited a reduced likelihood of experiencing symptomatic CNS progression when compared to their counterparts. These results collectively highlight the potent CNS protective effects demonstrated by alectinib.

Secondly, our findings indicate that approximately 30% of patients may experience oligo-progression upon resistance to ALK inhibitors. However, in real-world scenarios, local therapy was administered to only 50% of cases with oligo-progression, while clinicians commonly opted for a switch to second-generation ALK-TKIs following crizotinib-induced oligo-progression. In our research, we discovered that local treatment significantly extends the duration of previous targeted agents by approximately 6 months. Moreover, in accordance with evolutionary theory, complex mutation could be barely induced by local treatment. Therefore, it is imperative to prioritize the implementation of local therapy as an efficacious physical modality in clinical practice.

Moreover, despite the unanimous recommendation of repeated biopsy upon resistance to second-generation ALK-TKIs in all guidelines, our study revealed that only 50% of patients underwent this procedure in a real-world setting, potentially leading to suboptimal decisions regarding subsequent treatments. We also explored the effectiveness of second-generation ALK-TKIs following crizotinib resistance by presenting compelling cases. We harbored the idea that for patients experiencing extracranial progression following crizotinib resistance, re-biopsy and NGS may still be necessary as second-generation ALK-TKIs cannot offer a universal solution.

Finally, we conducted further investigations into the optimal treatment strategy following the progression of second-generation ALK-TKIs. Our research revealed a heterogeneous response to subsequent ALK-TKI treatment among different patients; specifically, patients harboring ALK resistance mutations demonstrated prolonged PFS and long-term OS compared to those without such mutations (this observation could be explained by the off-target theory in patients lacking secondary ALK mutations). In patients without secondary mutation in the ALK kinase domain following resistance to second-generation ALK-TKIs, there was no significant difference observed in the efficacy of chemotherapy or alternative ALK-TKI treatments. Notably, in a previous Phase II clinical trial, the ORR of lorlatinib was 32.1% in patients with one prior non-crizotinib ALK-TKI and 38.7% in patients with two or more prior ALK-TKIs [17]; although higher response rates were observed in patients harboring identified ALK mutations, lorlatinib demonstrated efficacy even in patients without identified mutations. And the option of chemotherapy with a platinum pemetrexed-based combination remains available after lorlatinib progression. Therefore, the selection of third-generation ALK-TKI or chemotherapy subsequent to resistance to second-generation ALK-TKI necessitates validation through standardized head-to-head studies. In this scenario, we currently recommend considering chemotherapy or third-generation ALK-TKIs based on the patient’s overall health status and treatment preferences. Furthermore, according to recent data from the CROWN study [15], first-line lorlatinib has achieved a 5-year PFS rate exceeding 60%, significantly surpassing the overall efficacy observed with the “2 + 3” pattern in our study. Consequently, lorlatinib should be strongly recommended for initial treatment of advanced ALK^+^ NSCLC; however, its long-term CNS adverse effects and cardiovascular toxicity resulting from hyperlipidemia necessitate evaluation over an extended duration.

In addition, our study had several limitations and a few questions remained unresolved. Firstly, this study was a retrospective study conducted at a single-center with limited sample size, thereby inevitably introducing selection biases. Secondly, in order to conduct a more comprehensive analysis, Cohort 1 required a larger sample size. Thirdly, further investigation is needed to fully discuss the optimal treatment strategy for patients who only experienced CNS progression after resistance to second-generation ALK-TKIs, including the selection of subsequent ALK inhibitors and determining the optimal timing for brain radiotherapy. Lastly, the utilization of diverse biopsy samples and NGS panels in our study may introduce potential sources of variability, thereby impacting the stability of the obtained results.

## Conclusions

First-line alectinib demonstrated superior efficacy in protecting the CNS compared to crizotinib. Clinicians should acknowledge the importance of repeated biopsy and local treatment. For patients who develop an ALK-resistant mutation following the resistance to second-generation ALK-TKIs, appropriate sensitive ALK-TKI should be administered. Whereas for those without such mutations, standardized head-to-head studies are needed to compare the efficacy of chemotherapy and third-generation ALK-TKIs, and before that, the selection of chemotherapy or third-generation ALK-TKI should be based on the patient’s overall physical health status and personal preferences.

### Electronic supplementary material

Below is the link to the electronic supplementary material.

Supplementary Material 1

Supplementary Material 2

## Data Availability

The datasets generated and analyzed during this study are available from the corresponding authors on reasonable request.

## Abbreviations

ALK: Anapastic lymphoma kinase
ALK-TKIs: ALK tyrosine kinase inhibitors
NSCLC: Non-small cell lung cancer
CNS: Central nervous system
ORR: Objective response rate
OS: Overall survival
ALK^+^: ALK-positive
BBB: Blood-brain barrier
NGS: Next-generation sequencing
EMR: Electronic Medical Records
RECIST 1.1: Response Evaluation Criteria in Solid Tumors version 1.1
PFS: Progression-free survival
HR: Hazard ratio
CI: Confidence interval
SCC: Squamous-cell carcinoma
RCTs: Randomized controlled trials
