# Supplementary material for: Progression patterns, resistant mechanisms and subsequent therapy for ALK-positive NSCLC in the era of second-generation ALK-TKIs
Source: J Transl Med. 2024 Jun 20;22:585. doi: 10.1186/s12967-024-05388-0 (PMC11191366; doi:10.1186/s12967-024-05388-0)

**Supplementary Fig. 1 | Flowchart depicting the enrollment of patients for analysis of second-generation ALK-TKI resistance.**


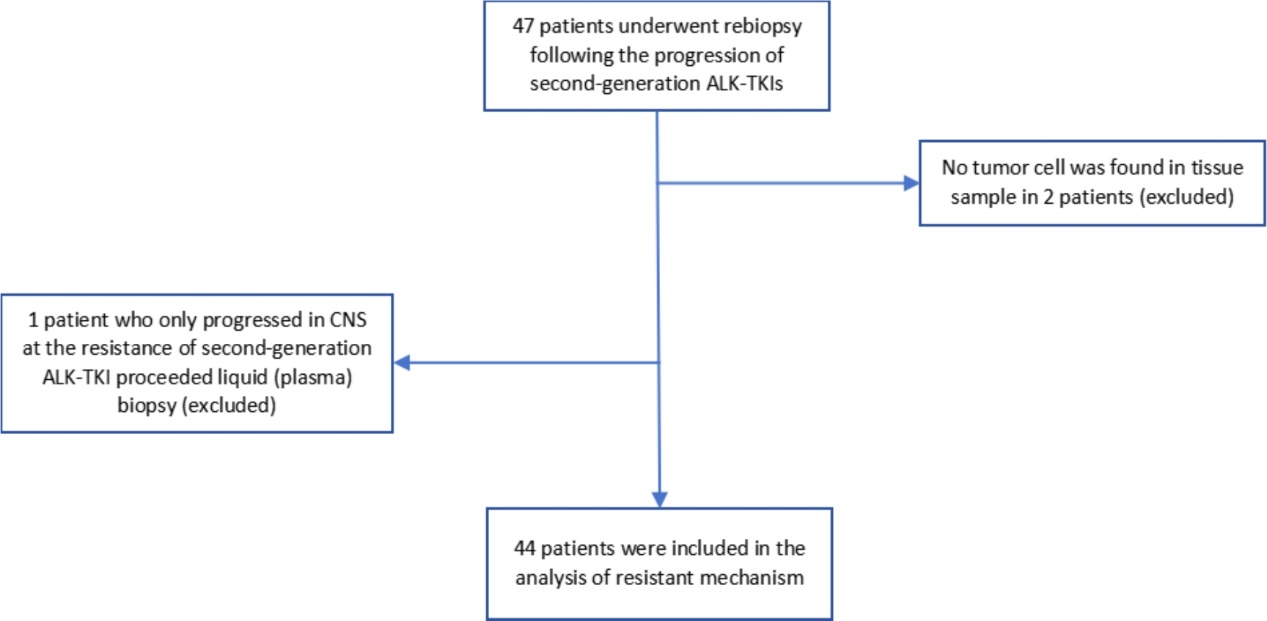

Supplement: Supplementary file 2 — Supplementary Material 2 [file 12967_2024_5388_MOESM2_ESM.docx]
