# Supplementary material for: Progression patterns, resistant mechanisms and subsequent therapy for ALK-positive NSCLC in the era of second-generation ALK-TKIs
Source: J Transl Med. 2024 Jun 20;22:585. doi: 10.1186/s12967-024-05388-0 (PMC11191366; doi:10.1186/s12967-024-05388-0)
Supplement: Supplementary file 1 — Supplementary Material 1 [file 12967_2024_5388_MOESM1_ESM.docx]

**Puyuan Xing, Professor**

Department of Medical Oncology, National Cancer Center/National Clinical Research Center for Cancer/Cancer Hospital, Chinese Academy of Medical Sciences and Peking Union Medical College

No 17 Panjiayuan Nanli, Chaoyang district, Beijing 100021, China.

Email: xingpuyuan@cicams.ac.cn

**Junling Li, Professor**

Department of Medical Oncology, National Cancer Center/National Clinical Research Center for Cancer/Cancer Hospital, Chinese Academy of Medical Sciences and Peking Union Medical College

No 17 Panjiayuan Nanli, Chaoyang district, Beijing 100021, China.

Email: lijunling@cicams.ac.cn

Dear editor,

We have enclosed here with a manuscript entitled “Progression patterns, resistant mechanisms and subsequent therapy for ALK-positive NSCLC in the era of second-generation ALK-TKIs” that we submit for your consideration for publication in your journal, *Journal of Translational Medicine*.

Since the identification of anaplastic lymphoma kinase (ALK) rearrangement in non-small cell lung cancer (NSCLC) in 2007, significant advancements have been made in this field. Patients with advanced ALK-positive (ALK^+^) NSCLC have experienced substantial survival benefits due to the development of multiple generations of ALK inhibitors. While clinical efficacy remains the primary endpoint in randomized controlled trials (RCTs), progression patterns, mechanisms of resistance, and subsequent therapies are not extensively discussed within this context. Here we followed two cohorts of patients and found that, for patients with ALK-resistant mutations following the resistance to second-generation ALK-TKIs, appropriate sensitive ALK-TKI should be administered; for those without such mutations, the selection of chemotherapy or third-generation ALK-TKI should be based on the patient's overall physical health and personal preferences. Our findings provide guidance for the posterior line therapy of patients with ALK^+^ NSCLC.

There are no conflicts of interest in the submission of this manuscript, and all authors have given their approval for its publication. The work has not been previously published, either in whole or in part, nor is it under consideration for publication elsewhere. All listed authors have endorsed the enclosed manuscript.

Thank you for your kind attention to our manuscript. We eagerly anticipate your response. Wish you all the best!

Sincerely,

Puyuan Xing

**April 23, 2024**
